# Development of anti-aflatoxin B1 nanobodies from a novel mutagenesis-derived synthetic library for traditional Chinese medicine and foods safety testing

**DOI:** 10.1186/s13036-023-00350-y

**Published:** 2023-04-24

**Authors:** Yu-Ching Lee, Gar-Hwa Lai, Tsai-Yu Lin, Tien-Sheng Tseng, Tsung-Hsun Tsai, Wang-Chuan Chen, Cheng-Chung Lee, Keng-Chang Tsai

**Affiliations:** 1 TMU Research Center of Cancer Translational Medicine, Taipei Medical University, Taipei, Taiwan; 2 Ph.D. Program for Cancer Molecular Biology and Drug Discovery, College of Medical Science and Technology, Taipei Medical University, Taipei, Taiwan; 3 Ph.D. Program in Drug Discovery and Development Industry, College of Pharmacy, Taipei Medical University, Taipei, Taiwan; 4 Ph.D. Program in Medical Biotechnology, College of Medical Science and Technology, Taipei Medical University, Taipei, Taiwan; 5 Department of Orthopedics, Kaohsiung Veterans General Hospital, Kaohsiung, Taiwan; 6 Institute of Molecular Biology, National Chung Hsing University, Taichung, Taiwan; 7 Department of Psychiatry, Kaohsiung Armed Forces General Hospital, Kaohsiung, Taiwan; 8 The School of Chinese Medicine for Post Baccalaureate, I-Shou University, Kaohsiung, Taiwan; 9 Department of Chinese Medicine, E-Da Hospital, Kaohsiung, Taiwan; 10 The Ph.D. Program for Translational Medicine, College of Medical Science and Technology, Taipei Medical University, Taipei, Taiwan; 11 National Research Institute of Chinese Medicine, Ministry of Health and Welfare, No. 155-1, Sec. 2, Linong St., Beitou District, Taipei, 11221 Taiwan

**Keywords:** Nanobody, Phage display technology, Glove-like cavity, Aflatoxin B1, Nonhypervariable complementarity-determining region 4 loop

## Abstract

**Background:**

The main commercially available methods for detecting small molecules of mycotoxins in traditional Chinese medicine (TCM) and functional foods are enzyme-linked immunosorbent assay and mass spectrometry. Regarding the development of diagnostic antibody reagents, effective methods for the rapid preparation of specific monoclonal antibodies are inadequate.

**Methods:**

In this study, a novel synthetic phage-displayed nanobody Golden Glove (SynaGG) library with a glove-like cavity configuration was established using phage display technology in synthetic biology. We applied this unique SynaGG library on the small molecule aflatoxin B1 (AFB1), which has strong hepatotoxicity, to isolate specific nanobodies with high affinity for AFB1.

**Result:**

These nanobodies exhibit no cross-reactivity with the hapten methotrexate, which is recognized by the original antibody template. By binding to AFB1, two nanobodies can neutralize AFB1-induced hepatocyte growth inhibition. Using molecular docking, we found that the unique non-hypervariable complementarity-determining region 4 (CDR4) loop region of the nanobody was involved in the interaction with AFB1. Specifically, the CDR4’s positively charged amino acid arginine directed the binding interaction between the nanobody and AFB1. We then rationally optimized the interaction between AFB1 and the nanobody by mutating serine at position 2 into valine. The binding affinity of the nanobody to AFB1 was effectively improved, and this result supported the use of molecular structure simulation for antibody optimization.

**Conclusion:**

In summary, this study revealed that the novel SynaGG library, which was constructed through computer-aided design, can be used to isolate nanobodies that specifically bind to small molecules. The results of this study could facilitate the development of nanobody materials to detect small molecules for the rapid screening of TCM materials and foods in the future.

**Supplementary Information:**

The online version contains supplementary material available at 10.1186/s13036-023-00350-y.

## Introduction

Camelids produce unique heavy-chain-only antibodies, which lack light chains. The variable antigen-binding domains of such antibodies are called variable parts of the heavy chain of a heavy-chain antibody (VHH). Single-domain antibodies (sdAbs) with antigen-binding affinity were first introduced in 1989 and have been validated as effective antigen-binding fragments [1, 2]. Subsequently, sdAbs were referred to as nanobodies. Nanobodies have a low molecular weight ranging between 12 and 15 kDa, are easy to mass produce, have high specificity and affinity, and are more stable than general antibodies. In addition, they have low immunogenicity and thus facilitate antibody engineering for the design of multivalent complexes for clinical and diagnostic applications [3]. Regarding production, compared with full-length antibodies, a microbial expression system can produce higher yields of functional antigen-binding fragments (e.g., sdAbs), which allow for the rapid production of safe drugs in relatively high quantities and at relatively low cost. Many similar antibody fragments expressed using bacteria or yeast are currently under evaluation for their clinical trial application [4]. Because of their excellent penetration effect, nanobodies have a wide range of potential applications. For example, they are suitable for use as diagnostic reagents or imaging agents. In addition, nanobodies can be used alone in clinical medicine as antagonistic antibody drugs or in combination with small-molecule drugs for targeted therapy [5–7].

In general, normal antibodies bind to antigens and even low-molecular-weight compounds e.g., haptens) by the paratope between the variable domains of heavy and light chains [8]. However, heavy-chain-only nanobodies, which lack light chains, rely entirely on a single variable domain to recognize antigenic targets. Most structural analyses have revealed that compared with general antibodies, nanobodies have more concentrated and compact paratopes with smaller molecular surfaces. Most nanobodies have atypical complementarity-determining region (CDR) 1 and CDR2 structures that incorporate extended CDR3 loops. In addition, the framework regions (FRs) of nanobodies exhibit many structural variations [9]. A study found that the surface of interaction between the nanobody and the hapten can be large enough to generate high-affinity binding [10]. Therefore, nanobodies have the potential to be developed as diagnostics for haptens such as caffeine [11]. A notable discovery was made by Fanning and Horn, who used a grafting technique to prepare an antimethotrexate (anti-MTX) nanobody. Against the researchers’ expectations, the crystal structure of the complex exhibited a noncanonical binding site that involved MTX tunnelling under the CDR1 loop. The anti-MTX nanobody has been proven to bind to hapten MTX molecules through CDR4, a nonhypervariable loop that plays a key role in generating high-affinity interactions [12]. Although nanobodies provide only a relatively small surface for interaction (because they have only a heavy-chain variable region domain), they can still generate high-affinity and specific binding reactions to targets with low molecular weights.

Aflatoxins, which are genotoxic hepatocarcinogens, are abundant in nature and cannot be completely detoxified through metabolic detoxification. They accumulate in tissues and have a high correlation with the development of hepatocellular carcinoma [13]. Aflatoxins are produced by *Aspergillus flavus* and *Aspergillus parasiticus* and can be found in mouldy grains, such as traditional Chinese medicine (TCM) materials, functional foods, peanuts, and corn. Thin layer chromatography and high-performance liquid chromatography are common methods for detecting aflatoxins in food [14]. Aflatoxins are difuranocoumarin derivatives, and aflatoxin B1 (AFB1) is the most common and most toxic aflatoxin. In 1993, AFB1 was classified as a class 1 carcinogen by the International Agency for Research on Cancer of the World Health Organization. In addition, the United States Food and Drug Administration considers AFB1 an unavoidable contaminant in food. AFB1 can suppress the immune system and reduce the cell growth rate. Furthermore, intoxication with AFB1 concurrent with hepatitis B infection is associated with a high risk of hepatocellular carcinoma lesions [15, 16]. Metabolized by cytochrome enzyme P450, AFB1 is activated to become a hepatotoxic molecule that binds to hepatocyte DNA and forms DNA adducts. These DNA adducts interact with the guanine bases of hepatocyte DNA, causing the formation of mutational hot spots at codon 249 in the p53 tumour suppressor gene. Gene mutations resulting from incorrect base insertion during DNA replication can lead to the formation of cancerous cells [15, 17]. AFB1 has a molecular weight of only 312 Da and is a hapten. Characterized by their low molecular weight, haptens cannot stimulate immune responses. Therefore, a hapten lacks immunogenicity and possesses only reactogenicity, which enables it to bind specifically to the corresponding antibody [18]. Nonetheless, a research group developed a nanobody known as NB26, which has a high affinity for AFB1, through alpaca immunization; this group also established several diagnostic tests [19–21]. These results support the validity and feasibility of developing a nanobody for hapten diagnosis.

Instead of using the conventional method of biological immunization to construct antibody libraries, this study attempted to construct antibody libraries through computer-aided design. The anti-MTX nanobody, an established structure, was used in this study as a template. In addition, a unique nonhypervariable CDR4 configuration was added to incorporate amino acid mutations at interaction hot spots, leading to the construction of a synthetic nanobody library with considerable complexity. This library can be applied for the screening of haptens and small-molecule compounds. Without the time-consuming step of biological immunization, the aforementioned method can rapidly and effectively produce specific nanobodies for tests or therapeutic reagent development.

## Materials and methods

### Cell culture

The normal human embryonic liver cell line CL48 was cultured in Eagle’s minimum essential medium supplemented with 10% fetal bovine serum. The human hepatoblastoma cell line HepG2.2.15 derived from the hepatocellular carcinoma cell line HepG2 with stable expression of transfected hepatitis B virus was cultured in Eagle’s minimum essential medium supplemented with 10% fetal bovine serum, 2 mmol/L glutamine, 1% nonessential amino acid, and 1% sodium pyruvate. The cell lines were purchased from American Type Culture Collection (Manassas, VA, USA). The cultures were maintained at 37 °C in a humidified atmosphere of 5% CO_2_ and 95% air.

### Synthetic nanobody library construction

Phage display libraries were constructed following the oligonucleotide-directed mutagenesis procedure proposed by Kunkel [22]. For more detailed descriptions, please refer to the protocol developed by Sidhu and Weiss [23]. In brief, this study employed structural biology and computer-aided mining to select a released nanobody structural sequence as the template (derived from protein data bank [PDB]: 3QXV). The three-dimensional template had a glove-like concave configuration, which inspired us to design a novel synthetic phage-displayed nanobody Golden Glove (SynaGG) library. PDB: 3QXV is a llama CDR1-4 graft nanobody antibody in complex with MTX. We synthesized a template nanobody gene harbouring TAA stop codons at positions chosen for randomization and subcloned it into the pCANTAB5E phagemid (GE Healthcare Inc.). Then NNK degenerate primers were used to mutate codons specifying 8 positions on template nanobody CDRs or FRs. TAA stop codons strategically positioned on the parent phagemid ensured that only phagemids carrying degenerate codons would yield fusion proteins with the pIII protein on the bacteriophage surface. To prevent interference from the degenerate NNK-producing TAG stop codon, *Escherichia coli* strain ER2738 (a *sup*E strain with glutamine-inserting amber (UAG) suppressor tRNA) was employed for library amplification. TAG stop codons are suppressed by glutamine in ER2738. Thus, we transformed the constructed library DNA into the ER2738 through electroporation and calculated the transformed numbers to estimate the complexity of the library. The VCS-M13 (Stratagene Corp.) helper phage was added to the transformed culture to initiate recombinant phage production. The phage library was precipitated with 4% polyethyl glycol 8000 and 3% NaCl (w/v), resuspended in phosphate-buffered saline (PBS) containing 1% bovine serum albumin (BSA), and stored at 4 °C.

### Biopanning and nanobody expression

After constructing the synthetic nanobody library, we used a panning method to enrich and isolate nanobodies with the specific ability to bind with AFB1 in the library. First, an AFB1 and BSA conjugate (AFB1-BSA) purchased form Sigma-Aldrich Corporation was coated onto the wells of a microtiter plate at 4 °C overnight. AFB1-BSA of 5 μg/well was used as the coating for the first round of panning; the antigen coating concentration in the second to fourth rounds was lowered to AFB1-BSA of 1 μg/well. The next day, the AFB1-BSA was removed, and the well was blocked with 3% BSA at room temperature for 1 h. Subsequently, library phage particles (10^11^) were mixed with 3% BSA at a 1:1 ratio, and this mixture was then added to the well and incubated at room temperature for 2 h. Next, unbound phages were removed, and the well was washed 10 times through pipetting with PBS with 0.05% Tween 20 (PBST). Bound phages were eluted with 0.1 M HCl–glycine (pH 2.2)/0.1% BSA elution buffer and neutralized with 2 M Tris base buffer. The eluted phages were then used to infect *E. coli* strain ER2738 immediately for phage amplification. The amplified phages were precipitated and recovered according to a previously described method and used in the next round of panning [24]. The panning procedure was repeated four times. After panning, total library DNA was purified and transformed into *E. coli* strain TOP 10F′ (a nonsuppressor strain; Invitrogen). The transformed clones were randomly selected for individual nanobody expression and subsequently for binding analysis by ELISA. The positive clones were sequenced to infer the nanobodies’ primary structure. The nanobody genes were fused with HA and His tags. For further nanobody protein expression and purification, the interesting clone was grown overnight in 0.5 mM isopropyl b-D-thiogalactopyranoside (IPTG) for nanobody induction. Subsequently, recombinant nanobodies were purified with Ni^2+^-charged sepharose according to the manufacturer’s instructions (GE Healthcare Inc.).

### Sequence analysis

To sequence the nanobody clones of interest, we used a primer (5′-GCTATGACCATGATTACGCCA-3′) complementary to the pectate lyase B signal sequence placed before the heavy chain variable region. Next, the international ImMunoGeneTics (IMGT) information system/V-QUEry and standardization system (http://imgt.org) were used to compile and analyse the sequence data.

### Enzyme-linked immunosorbent assay

AFB1-BSA, MTX-BSA, or AFB1–ovalbumin (OVA) (0.5 μg/well) was coated onto the wells of a microtiter plate at 4 °C overnight. These wells were blocked with 5% skimmed milk, and nanobodies were then added to the wells at room temperature for 1 h. After a wash with PBST, bound nanobodies were detected, and signals were developed using the horseradish peroxidase (HRP)–conjugated mouse anti-HA tag antibody (Cell Signaling Technology, Inc.). Finally, 3,3′,5,5′-tetramethylbenzidine dihydrochloride (TMB) was added for signal development. The reaction was stopped through the addition of 1 N HCl, and absorbance was determined through optical density (OD) measurement at 450 nm.

### Competitive inhibition assay

Competitive inhibition assays were employed to determine the binding specificity of AFB1-binding nanobody molecules. In brief, microtiter plates were coated with AFB1-OVA at 0.5 μg/well and blocked with 5% skimmed milk. After 1 h of incubation at room temperature, the plates were washed with PBST. The purified nanobody of 10 μg/mL was incubated first with multiple concentrations of soluble free AFB1 diluted in ddH_2_O (ranging from 1.6 to 100 ng/mL) at room temperature for 1 h. Then, the mixtures were added to the wells to react with the coated AFB1-OVA molecule. After incubation at room temperature for 1 h, the plates were washed with PBST. Subsequently, the HRP-conjugated mouse anti-HA tag antibody (Cell Signaling Technology, Inc.) was added at room temperature for 1 h to detect bound nanobodies. After a wash with PBST, TMB substrate was added to each well for development. The reaction was stopped with 1 N HCl, and signal intensity was measured through OD measurement at 450 nm.

### Cell proliferation assay

Cell proliferation was measured using the 3-(4,5-dimethylthiazol-2-yl)-5-(3-carboxymethoxyphenyl)-2-(4-sulfophenyl)-2H-tetrazolium (MTS) Cell Proliferation Assay Kit (Promega). HepG2.2.15 or CL48 cells were seeded at a density of 5000 cells/well in a 96-well culture plate. AFB1 was mixed with or without different concentrations of nanobodies and incubated at room temperature for 30 min. The mixture was then added to the cell culture and incubated for 48 h. Finally, an MTS and phenazine methosulphate mixture was added and incubated for 90 min for development. After the sodium dodecyl sulphate (SDS) reagent was added to stop the reaction, the absorbance of each well was determined through OD measurement at 490 nm.

### Molecule modelling and molecular docking

To investigate how the candidate nanobodies A1, F2, and A1F2 interacted with AFB1, homology modelling was employed to create a three-dimensional structure of the nanobody with PDB: 3QXV as the template in BIOVIA Discovery Studio (Dassault Systèmes, BIOVIA Corp., San Diego, CA, USA). The crystal structure of the template VHH antibody was downloaded from the Research Collaboratory for Structural Bioinformatics Protein Data Bank. The interaction between proteins and the AFB1 compound was simulated using the CHARMm force field. The AFB1 compound was docked into the CDR-H loop of the nanobody with the GOLD docking tool. The docking parameter settings are described as follows: The MTX structure of the VHH template (PDB: 3QXV) was set as the centre to define the binding site sphere (10 Å). The GOLD tool with a genetic algorithm was used to simulate AFB1 compound docking into the CDR pocket of a nanobody with a flexible state. In total, 200 docking runs were generated for each AFB1 molecule to simulate the interaction of AFB1 with the CDR binding site of the nanobody. These conformations were then further subjected to automatic methods to identify the optimal AFB1–nanobody interaction configurations. During the simulation, the cation-pi interaction, hydrophobic interaction, and ligand torsion strain were accounted for in the CHEMPLP score function.

### Statistical analysis

All data were analysed using GraphPad Prism (GraphPad, CA, USA) and are presented as mean ± standard error of the mean. Statistical comparisons between groups were performed using a one-way analysis of variance followed by post hoc analyses with the Tukey HSD protected least significant difference test. *P* values lower than 0.05 were considered significant.

## Results

### The experimental flowchart for generating specific nanobody

**Fig. 1:**
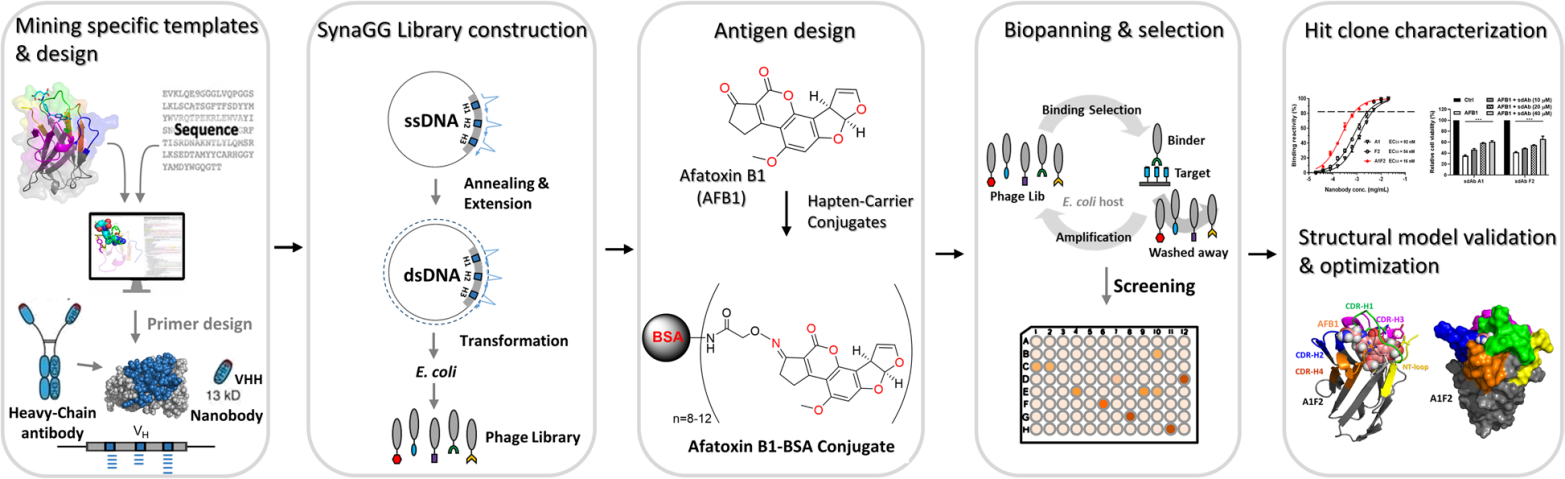
Experimental flowchart for isolating specific nanobodies based on molecular structure. This study used a nanobody-small molecule complex structure that had been published previously as the foundation of the experiment to design a synthetic library. Subsequently, carrier protein–conjugated AFB1 was used for panning to isolate specific nanobodies for analysis, validation, and optimization

We employed structural methods to establish an effective and rapid antibody panning platform. By targeting small molecules or haptens with low immunogenicity, the SynaGG library was constructed to isolate specific nanobodies for clinical and diagnostic applications. Figure 1 presents a flowchart of the experiment. We used published structural sequences for design and modification, applied site-direct mutagenesis to construct the SynaGG library, and conducted panning of the designed antigen on the phage display platform to isolate nanobodies that specifically bound to small molecules. Subsequently, the isolated nanobodies were characterized. In addition, antibody engineering was used to verify the molecular docking analysis and optimize the design.

### Rational design of a favourable small-compound synthetic nanobody library

**Fig. 2:**
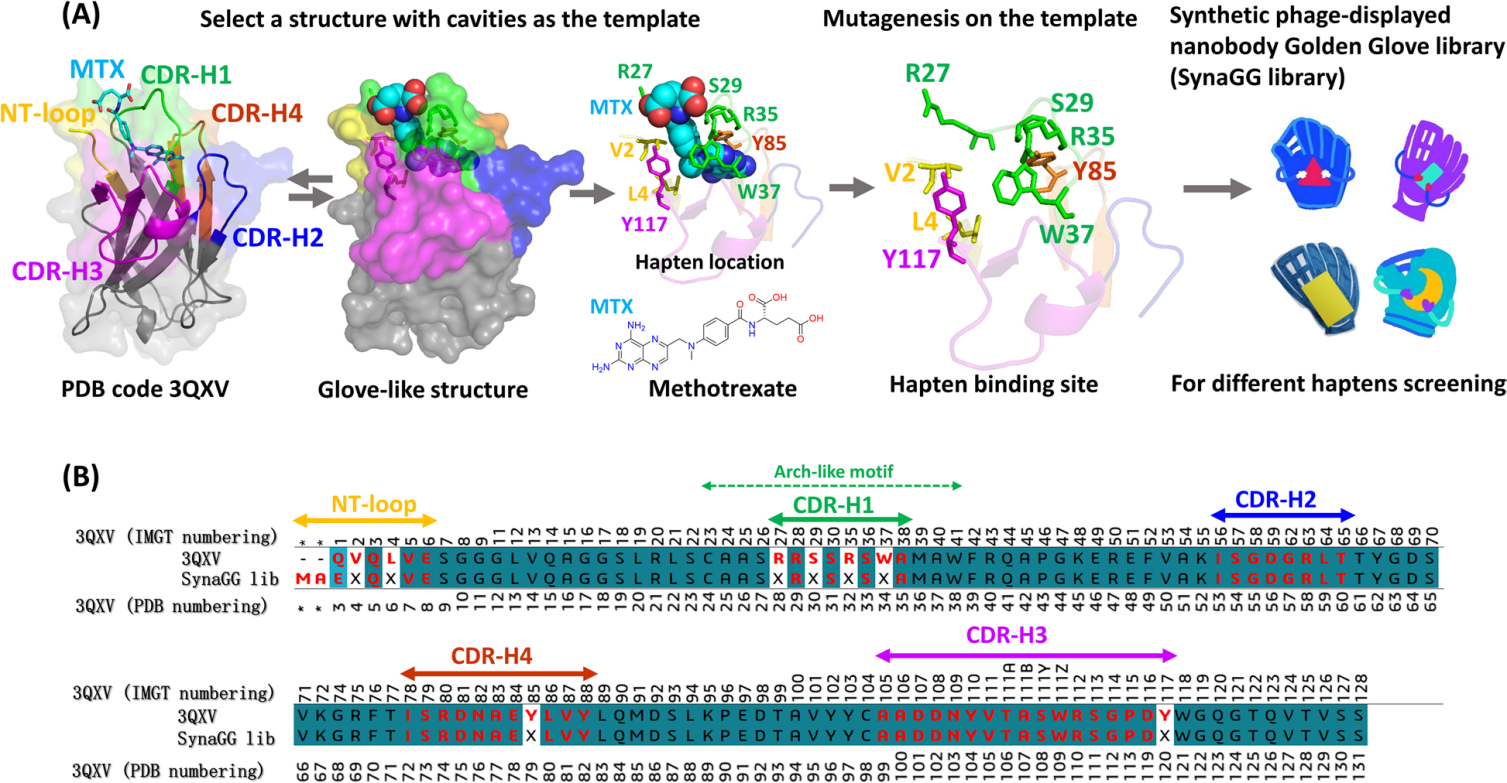
Complex structure of PDB: 3QXV used to design a synthetic phage-displayed nanobody library. **A** In the structure of PDB: 3QXV, key amino acids that interact with MTX in the nanobody were analysed. Positions NT, CDR-H1, CDR-H2, CDR-H3, and CDR-H4 are marked in different colours. The SynaGG library was designed using the nanobody based on the PDB: 3QXV structure. Mutated nanobody molecules in the SynaGG library demonstrated a glove-like configuration, which was suitable for panning small molecules. **B** The red parts in the figure denote the positions of NT, CDR-H1, CDR-H2, CDR-H3, and CDR-H4, including mutation points in the nanobody SynaGG library. The IMGT numbers represent the unique IMGT numbering, denoting the positions of the amino acids. NT-loop represents the nanobody N-terminus position, and X represents the NNK mutation

We used a published complex structure (PDB: 3QXV) to design a favourable small-compound synthetic nanobody library. PDB: 3QXV is a complex structure combining a llama nanobody and the small compound MTX. This structure is shown in Fig. 2A. The key amino acids of nanobodies that interacted with MTX were V2 and L4 of the N-terminus FR and R27, S29, R35, and W37 of CDR1. One crucial interaction was directed by CDR1, whereas the other two crucial interaction points were Y117 of CDR3 and Y85 of nonhypervariable CDR4. During interaction, the amino acids surrounded MTX, forming a glove-like structure that, in the opinion of us, was suitable for binding small molecules. That is, the nanobody interaction site that resembled a glove could stably catch small molecules (represented as balls). Therefore, using primers, we mutated the amino acids at the aforementioned positions (NNK codon) to construct the SynaGG library; the locations of these mutations in the library are shown in Fig. 2B. Next, Fig. 2B presents the amino acid numbering of the nanobody template defined on the IMGT scheme and the number corresponding to PDB. After transforming the constructed library DNA into *E. coli* ER2738, we calculated the library complexity as 8.3 × 10.^9^ To confirm the quality of the SynaGG library, 250 sequences in the library were randomly selected for sequencing. It was found that 238/250 (95.2%) of the sequences were in frame for the complete inserted nanobody gene. The 58 sequences with complete NNK substitution on 8 positions after the exclusion of NNK partial replacement sequences (parent template with TAA codons in the mutagenesis positions) are shown in Supplementary Fig. S1 to illustrate the diversity of the library. These results support the feasibility of using the SynaGG library for panning.

### Isolation of specific anti-AFB1 nanobody through panning

**Fig. 3:**
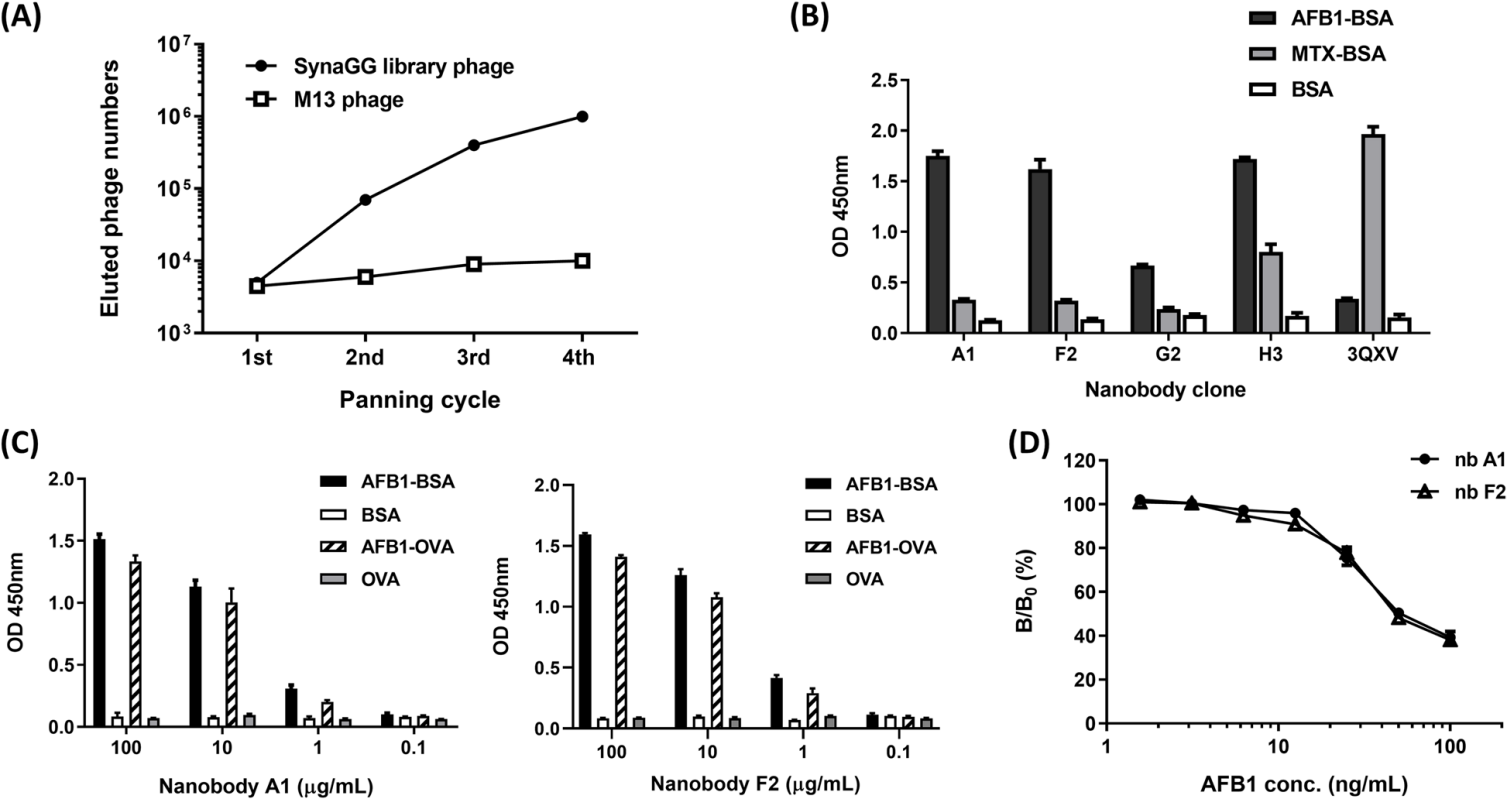
Characterization of anti-AFB1 nanobodies isolated from SynaGG library. **A** The eluted number of anti-AFB1 library phages was calculated after each round of panning. The wild-type M13 phage was a negative control. **B** Test of the binding reactivity of four representative nanobodies at the same concentration of 10 μg/mL to AFB1-BSA and MTX-BSA. BSA was used as a negative antigen control, and 3QXV denotes the original template nanobody that can recognize MTX. **C** Serial dilution on the two nanobodies (left: A1; right: F2) to test the binding reactivity to AFB1-BSA and AFB1-OVA. **D** A competitive inhibition assay determined the binding specificity of the nanobodies (nb A1 and nb F2) against the AFB1 molecule. The amount of bound nanobody in the presence of a free AFB1 inhibitor was measured and expressed as a percentage of the binding of the nanobody in the absence of an inhibitor. B and B_0_ denote the amount of bound nanobody in the presence and absence of the inhibitor, respectively

By using phage display technology, we constructed the SynaGG library to isolate specific anti-AFB1 nanobodies. The experimental results are presented in Fig. 3A. In the SynaGG library, the binding phage numbers increased by a factor of approximately 200 from the first round to the fourth round of panning. No meaningful change was observed when the control wild-type M13 phage was used for panning. In addition, we employed phage ELISA to test the binding reaction of amplified phage population following each selection round to the antigen AFB1-BSA. The phage library exhibited AFB-1 binding phage enrichment after the second round of panning (supplementary Fig. S2A). The SynaGG library was enriched in specific clones after the second round of panning. The amino acid mutations designed in the library led to the development of nanobodies that could interact with the antigen AFB1. In the single colony analysis for randomly selected sdAb clones, although some sdAb clones exhibited cross-reactivity to BSA, our results also demonstrated the isolation of AFB-1 specific binding clones (supplementary Fig. S2B). After gene sequencing, we selected four representative nanobodies. These four nanobody clones were induced by IPTG for protein expression and subsequent purification. SDS–polyacrylamide gel electrophoresis was then employed to analyse the purified nanobody protein, as illustrated in Supplementary Fig. S3. The purified nanobody exhibited a significant major band at a predicted molecule weight of approximately 15 kDa. Using an enzyme-linked immunosorbent assay (ELISA), we tested whether the isolated anti-AFB1 nanobodies would cross-react with small molecular MTX, which was recognized by the original template nanobody. The experimental results are provided in Fig. 3B. The original template nanobody 3QXV recognized the MTX molecule, whereas nanobodies A1 and F2 specifically recognized AFB1. The binding reaction of nanobody G2 was weak, and nanobody H3 had a cross-reaction with small molecular MTX. We tested the specificity of the isolated nanobodies A1 and F2 in relation to AFB1. Using AFB1-OVA as the antigen, we found that both isolated nanobodies could recognize AFB1-OVA in a dose-dependent manner (Fig. 3C). Moreover, it was confirmed that free AFB1 could compete for nanobody binding to AFB1-OVA with a competitive ELISA (Fig. 3D). These data evidenced that isolated nanobodies could specifically bind to AFB1 molecules.

### Anti-AFB1 nanobody can neutralize AFB1-induced liver cell growth inhibition

**Fig. 4:**
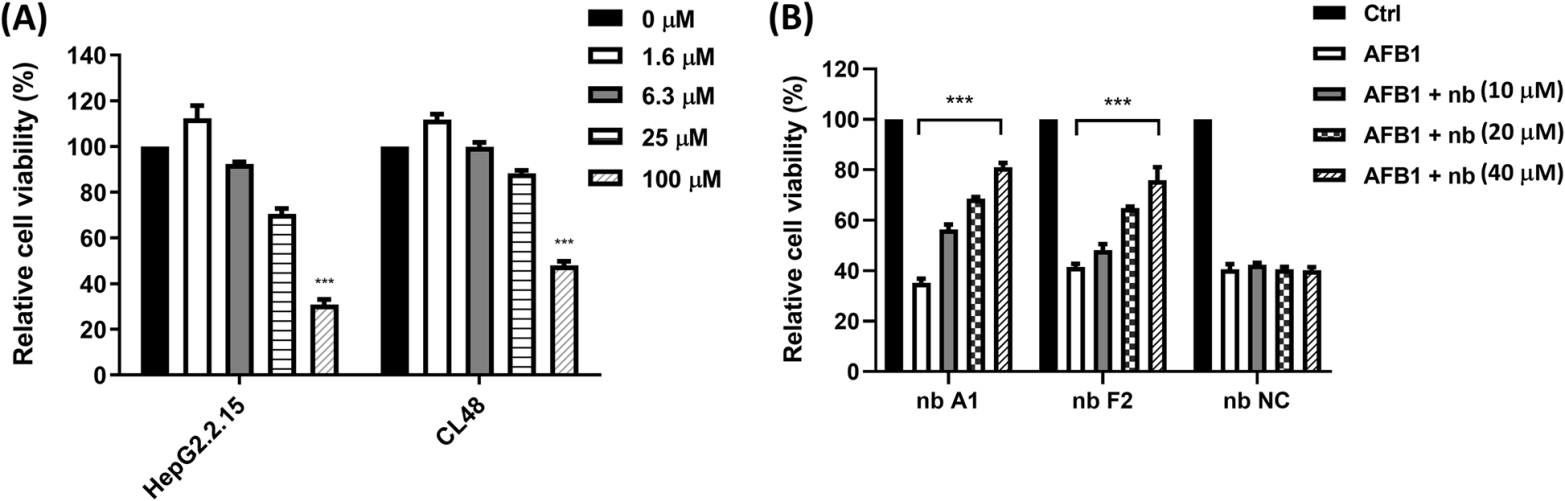
Isolated nanobodies can neutralize AFB1-induced hepatocyte growth inhibition by binding to AFB1. **A** The growth inhibitory effect of AFB1 at multiple concentrations in CL48 normal human liver cells and HepG2.2.15 human hepatoblastoma cells was measured after 48 h of treatment. **B** Nanobodies A1 (nb A1) and F2 (nb F2) interacted with AFB1 at a specified concentration; the protective effect on cell growth was monitored after treating HepG2.2.15 cells with AFB1 for 48 h; ***P < 0.001. An irrelevant nanobody (nb NC) that did not recognize AFB1 was used as the negative control

AFB1 is cytotoxic; thus, AFB1 accumulation due to prolonged intake can interfere with cell growth and induce cytopathic effects. The results of the experiment using hepatocytes to assay cytotoxicity are presented in Fig. 4A. The growth of both CL48 normal human liver cells and HepG2.2.15 human hepatoblastoma cells was significantly inhibited after treatment with 100 μM highly concentrated AFB1 for 48 h, and a dose-dependent effect was observed. In the subsequent experiment, we used the isolated nanobodies A1 and F2 to test whether the AFB1-induced cytostatic response could be neutralized by the specific binding of the nanobody to the small molecular AFB1. Based on the findings presented in Fig. 4A, 100 μM AFB1 was used to induce HepG2.2.15 cell growth inhibition. Compared with the irrelevant nanobody without neutralization ability, the AFB1-induced cytostatic response weakened alongside an increase in the indicated nanobody concentration, exhibiting a dose-dependent effect (Fig. 4B). At a maximum nanobody concentration of 40 μM, the AFB1-induced cytostatic response was weakened by approximately 70%.

### Molecular docking of nanobodies with AFB1

**Fig. 5:**
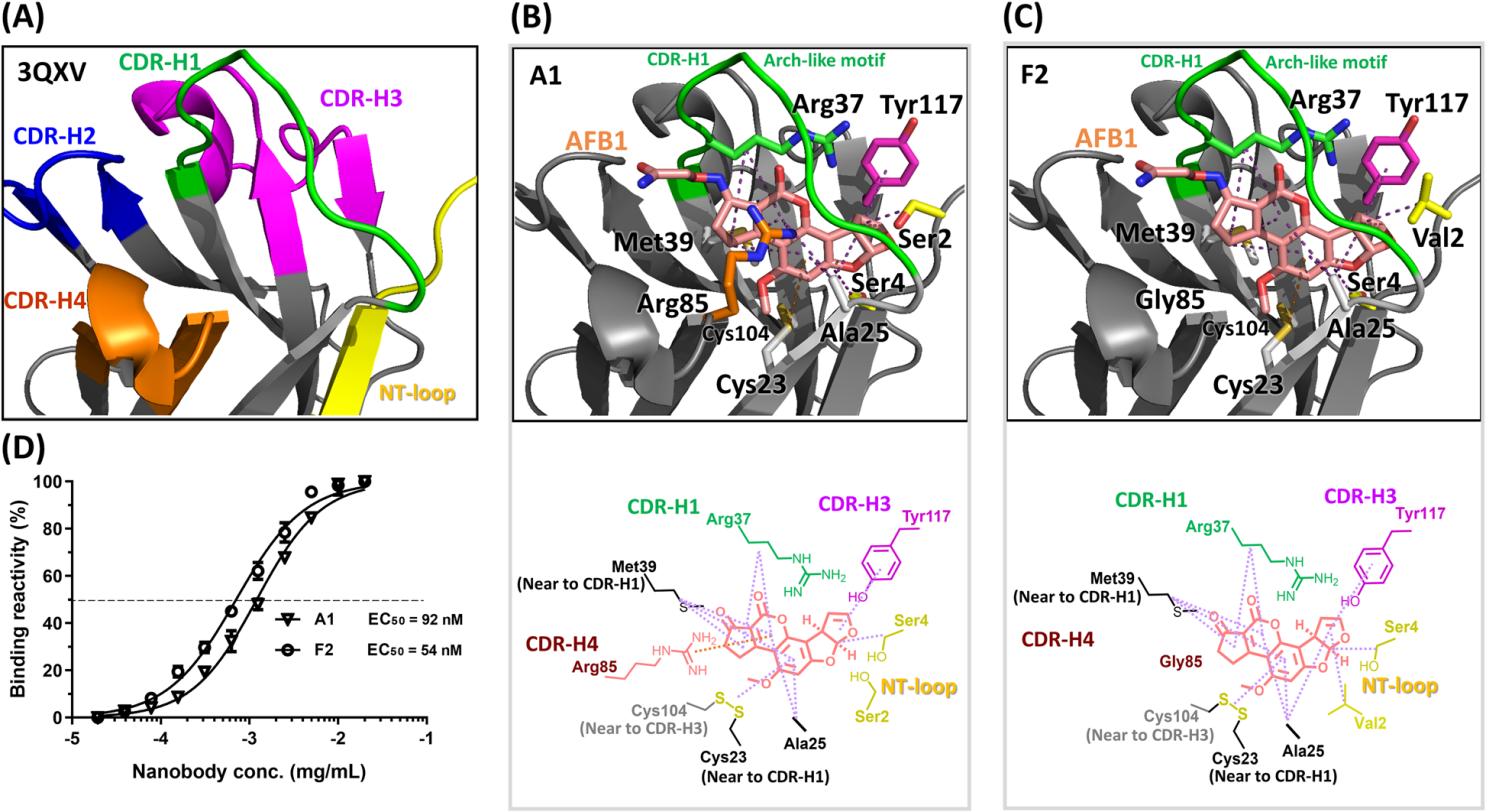
Interaction of nanobodies A1 and F2 with AFB1. Molecular docking was used to predict the interaction between the key residues of nanobodies A1 and F2 with AFB1. **A** The structure of the template nanobody 3QXV is shown by the cartoon diagram, including CDR-H1 (green), CDR-H2 (blue), CDR-H3 (magenta), CDR-H4 (orange), and N-terminal (NT) (yellow) loops. **B, C** Schematic representing all interactions between individual nanobodies A1(B) and F2(C) with AFB1. The interacting residues of a nanobody with AFB1 are present in the panel. The AFB1 molecule is shown as a stick model, and its carbon atoms are in pink. The interactions between the AFB1 molecule and the nanobody residues are represented by orange and magenta dotted lines, indicating cation-pi interaction and hydrophobic interactions, respectively. **D** The percentages of the binding of the serially diluted A1 and F2 nanobodies to AFB1 were calculated. The dashed lines indicate the nanobody concentration at EC_50_

**Fig. 6:**
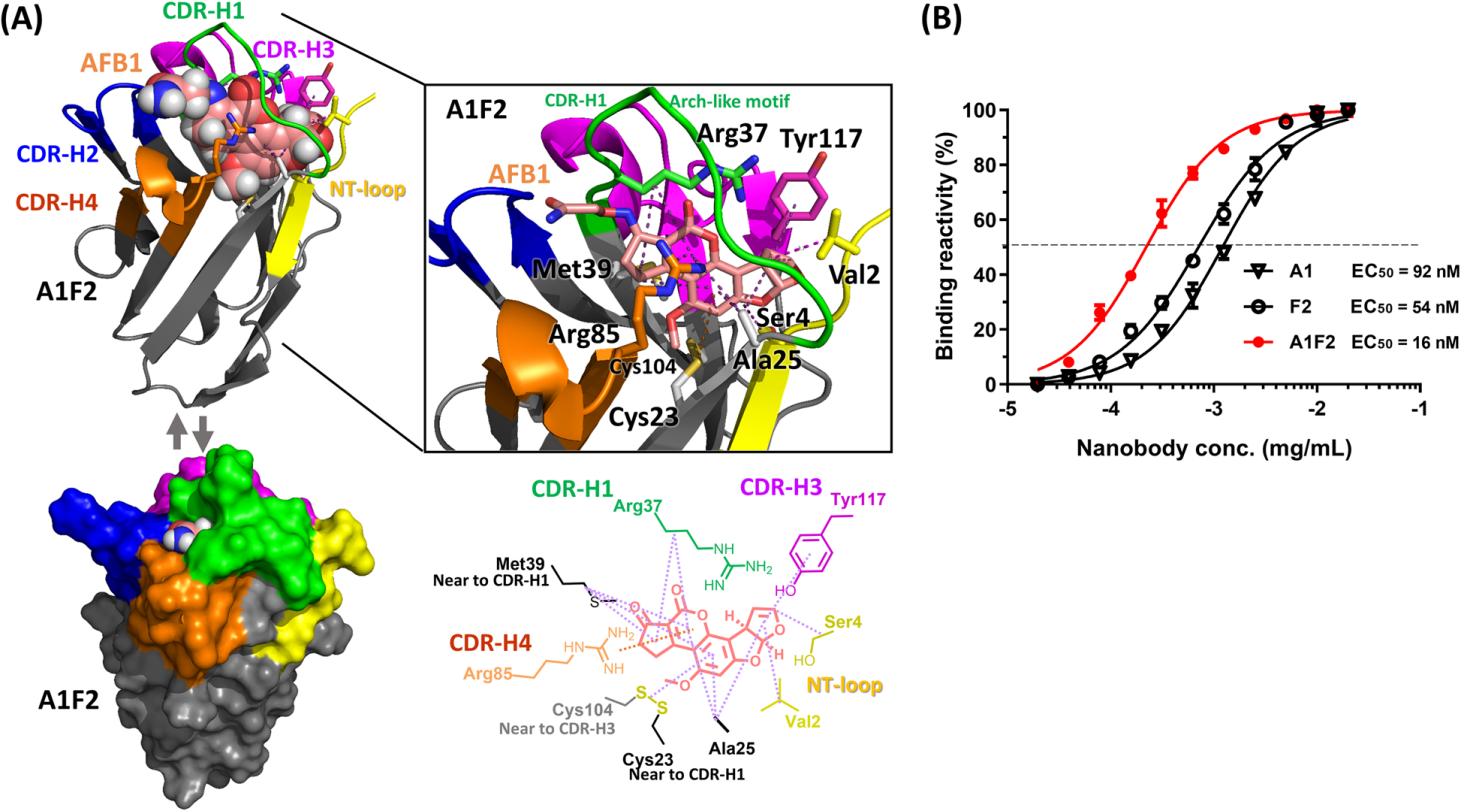
Effect of modifying the hot spot residue of nanobody A1F2 on the interaction with AFB1. **A** Effect of mutated nanobody A1F2 (cartoon and surface) on its interaction with AFB1; CDRs are marked in different colours. **B** The percentage of the binding of the serially diluted nanobody mutant A1F2 to AFB1 is denoted by the red line solid circles for comparison with the responses of nanobody A1 (black line inverted hollow triangles) and nanobody F2 (black line hollow circles). The dashed lines indicate the nanobody concentration at EC_50_

The structure of the template nanobody 3QXV including CDR-H1, CDR-H3, CDR-H4, and the N-terminal loop were designed to interact with haptens (Figs. 2A and 5A). The molecular modelling of the AFB1 in the binding pocket surrounded by nanobody A1 (Fig. 5B) indicated that the AFB1 moiety exhibited extensive hydrophobic interactions with a Cys23/Cys104 disulphide bond and four residues (Arg37, Met39, Ala25, and Ser4). The AFB1 forms cation-pi and pi-pi interactions with Arg85 and Tyr117, respectively. Nanobody F2 (Fig. 5C) exhibited one more hydrophobic interaction than did nanobody A1, namely that between AFB1 and Val2; however, nanobody F2 also lacked a cation-pi interaction with Gly85. The binding affinity for AFB1 was tested through serial dilution of the nanobody; the half maximal effective concentration (EC_50_) values of nanobodies A1 and F2 were calculated to be 92 and 54 nM, respectively (Fig. 5D). Based on the structural interaction, we explored whether modifying the hot spot residue of a nanobody could optimize its binding affinity. For this purpose, we constructed nanobody A1F2 to test whether mutating the serine at position 2 from amino acid into valine would improve the binding affinity of the nanobody to AFB1. Nanobody A1F2 exhibited all the interactions of nanobody A1 as well as one hydrophobic interaction, namely that of Val2 with AFB1. These results are shown in Fig. 6A. At position 2 of the nanobody sequence, mutating the amino acid from serine to valine enabled one more hydrophobic interaction, namely that between the AFB1 and Val2 of nanobody F2, which improved the affinity. We estimated the EC_50_ through the binding reaction of the serially diluted nanobody A1F2. The binding affinity for AFB1 increased by a factor of 5.75 from nanobody A1 to mutated nanobody A1F2; specifically, the binding affinity values of nanobody A1 and nanobody A1F2 were 92 and 16 nM, respectively (Fig. 6B). This result supported the feasibility of using molecular modelling for antibody engineering design.

## Discussion

We employed the nanobody structure in PDB that binds to small molecules as a template and used computer-aided analysis to design mutation points in order to construct a unique synthetic nanobody library. Through panning, nanobodies that specifically bind to AFB1 were isolated. The increase in the binding phage numbers was consistent with the results of our previous study, which involved the construction of a library based on animal immunization for panning [25]. Thus, nanobodies have great potential for binding to low-molecular-weight targets, and the proposed panning platform can successfully isolate nanobodies that bind to small molecules. However, compared with a natural repertoire antibody library prepared from an immunized animal, a synthetic library, where the panning process is based on the degree of affinity, is associated with increased difficulty and uncertainty. Therefore, the principle of absorption was applied in the panning process of this study to eliminate the possibility of nonspecific binding strains being selected. The position of the nonhypervariable CDR4 loop in the isolated nanobody plays a crucial role in antigen binding. Based on the present experiments, mutating the position (Ser2Val) can increase the binding affinity of nanobodies for AFB1 (Fig. 6). In addition, the presence of unique regions in isolated nanobodies increased variability, enabling nanobodies to recognize the structure of small molecules; the added interaction areas were key to nanobodies’ binding affinity and specificity in relation to AFB1.

The primary purpose of using a synthetic library is to customize the library to meet the screening requirements and to improve the experimental design based on the obtained information [7, 26]. In the future, we will optimize the design of the second-generation library by referring to amino acid properties to generate mutagenesis. When generating antibodies using animal immunization, ineffective antibody reactions may occur due to factors such as lack of antigenicity or predominant epitope structure. Synthetic library advantages can provide solutions to these problems. In this study, all mutations occurred on the side of the antigen-binding loops of the nanobody in the design of crucial mutation points on the synthetic library. Based on the structure, we found that CDR-H1 on the nanobody of 3QXV can affect the shape of the hapten entry through amino acid mutagenesis. In the design, amino acids 23-26 and 39-41 are two beta-sheets connecting CDR-H1 to form an arch-like motif. As shown in Figs. 2A and 6, we found that the hapten (AFB1) is embedded into the binding sites. Therefore, we use the term “glove” to illustrate the potential of the SynaGG library to isolate specific nanobodies to recognize (catch) different haptens (balls).

Because of its small structure, a nanobody can utilize the relatively long CDR3 loop to bind to deep antigen structures and thus generate a strong penetration effect. However, for nanobodies that bind to low-molecular-weight targets, intermolecular interactions occur among a small number of interfacial amino acids. Therefore, increasing the area of interaction by incorporating a nonhypervariable region is advantageous for hapten binding. In addition, increasing the number of hot spots plays a crucial energetic role in molecular recognition. We expected that using the mutation or length variation of the nonhypervariable CDR4 loop to design a synthetic nanobody library would facilitate the construction of a library of nanobodies with a wider binding surface and thus enable binding to more diverse hapten configurations. However, the nonhypervariable CDR4 loop, which produced additional interactions of the nanobody, may form traps in humanization and neglect key interactions and thus could affect the CDR grafting results. This problem must be addressed in relation to the performance of antibody engineering. In summary, the results of this study could provide valuable insight into interactions between nanobodies and haptens and thus facilitate further exploration.

## Supplementary Information

**Additional file 1: Supplementary Fig. S1.** The 58 sequences with complete NNK substitution on 8 mutagenesis positions in the SynaGG library. **Supplementary Fig. S2.** Amplified library phage ELISA and single colony analysis. **Supplementary Fig. S3.** The purified nanobody proteins were analyzed by SDS-PAGE. Please check additional file if captured correctly. Correct, but the fourth affiliation needs to be revised to match the manuscript. Revise from: The Ph.D. Program for Medical Biotechnology, College of Medical Science and Technology To: Ph.D. Program in Medical Biotechnology, College of Medical Science and Technology.

## Data Availability

All data generated or analyzed during this study are included in this published article.

## Abbreviations

AFB1: Aflatoxin B1
BSA: Bovine serum albumin
CDR: Complementarity-determining region
EC_50_: Half maximal effective concentration
ELISA: Enzyme-linked immunosorbent assay
FR: Framework regions
HRP: Horseradish peroxidase
IPTG: Isopropyl b-D-thiogalactopyranoside
IMGT: ImMunoGeneTics
MTS: 3-(4,5-dimethylthiazol-2-yl)-5-(3-carboxymethoxyphenyl)-2-(4-sulfophenyl)-2H-tetrazolium
MTX: Methotrexate
OD: Optical density
OVA: Ovalbumin
PBS: Phosphate-buffered saline
PBST: PBS with 0.05% Tween 20
PDB: Protein data bank
sdAb: Single-domain antibody
SDS: Sodium dodecyl sulphate
SynaGG: Synthetic phage-displayed nanobody Golden Glove
TCM: Traditional Chinese medicine
TMB: 3,3′,5,5′-tetramethylbenzidine dihydrochloride
VHH: Variable parts of the heavy chain of a heavy-chain antibody
