# Supplementary material for: Development of anti-aflatoxin B1 nanobodies from a novel mutagenesis-derived synthetic library for traditional Chinese medicine and foods safety testing
Source: J Biol Eng. 2023 Apr 24;17:30. doi: 10.1186/s13036-023-00350-y (PMC10127376; doi:10.1186/s13036-023-00350-y)

**Supplementary Material**

**Development of anti-aflatoxin B1 nanobodies from a novel mutagenesis-derived synthetic library for traditional Chinese medicine and foods safety testing**

**Yu-Ching Lee^1,2,3,4,#^, Gar-Hwa Lai^5#^, Tsai-Yu Lin^2^, Tien-Sheng Tseng^6^, Tsung-Hsun Tsai^7^, Wang-Chuan Chen^8,9^, Cheng-Chung Lee^10^, Keng-Chang Tsai^4,11,*^**

^1^TMU Research Center of Cancer Translational Medicine, Taipei Medical University, Taipei, Taiwan

^2^Ph.D. Program for Cancer Molecular Biology and Drug Discovery, College of Medical Science and Technology, Taipei Medical University, Taipei, Taiwan

^3^Ph.D. Program in Drug Discovery and Development Industry, College of Pharmacy, Taipei Medical University, Taipei, Taiwan.

^4^The Ph.D. Program for Medical Biotechnology, College of Medical Science and Technology, Taipei Medical University, Taipei, Taiwan.

^5^Department of Orthopedics, Kaohsiung Veterans General Hospital, Kaohsiung, Taiwan.

^6^Institute of Molecular Biology, National Chung Hsing University, Taichung, Taiwan

^7^Department of Psychiatry, Kaohsiung Armed Forces General Hospital, Kaohsiung, Taiwan.

^8^The School of Chinese Medicine for Post Baccalaureate, I-Shou University, Kaohsiung, Taiwan.

^9^Department of Chinese Medicine, E-Da Hospital, Kaohsiung, Taiwan.

^10^The Ph.D. Program for Translational Medicine, College of Medical Science and Technology, Taipei Medical University, Taipei, Taiwan

^11^National Research Institute of Chinese Medicine, Ministry of Health and Welfare, Taipei, Taiwan.

^#^These authors contributed equally to this work.

*Address for correspondence:

Keng-Chang Tsai, Ph.D.,

National Research Institute of Chinese Medicine, Ministry of Health and Welfare

No. 155-1, Sec. 2, Linong St., Beitou District, Taipei 11221, Taiwan

Tel: +886-2-28201999 ext 6241

E-mail: tkc@nricm.edu.tw

**Supplementary Figure S1. The 58 sequences with complete NNK substitution on 8 mutagenesis positions in the SynaGG library.** The red parts in the figure denote the positions of NT, CDR-H1, CDR-H2, CDR-H3, and CDR-H4, including mutation points in the nanobody SynaGG library. Q with the red background represents TAG stop codons suppressed by glutamine in *E. coli* strain ER2738.


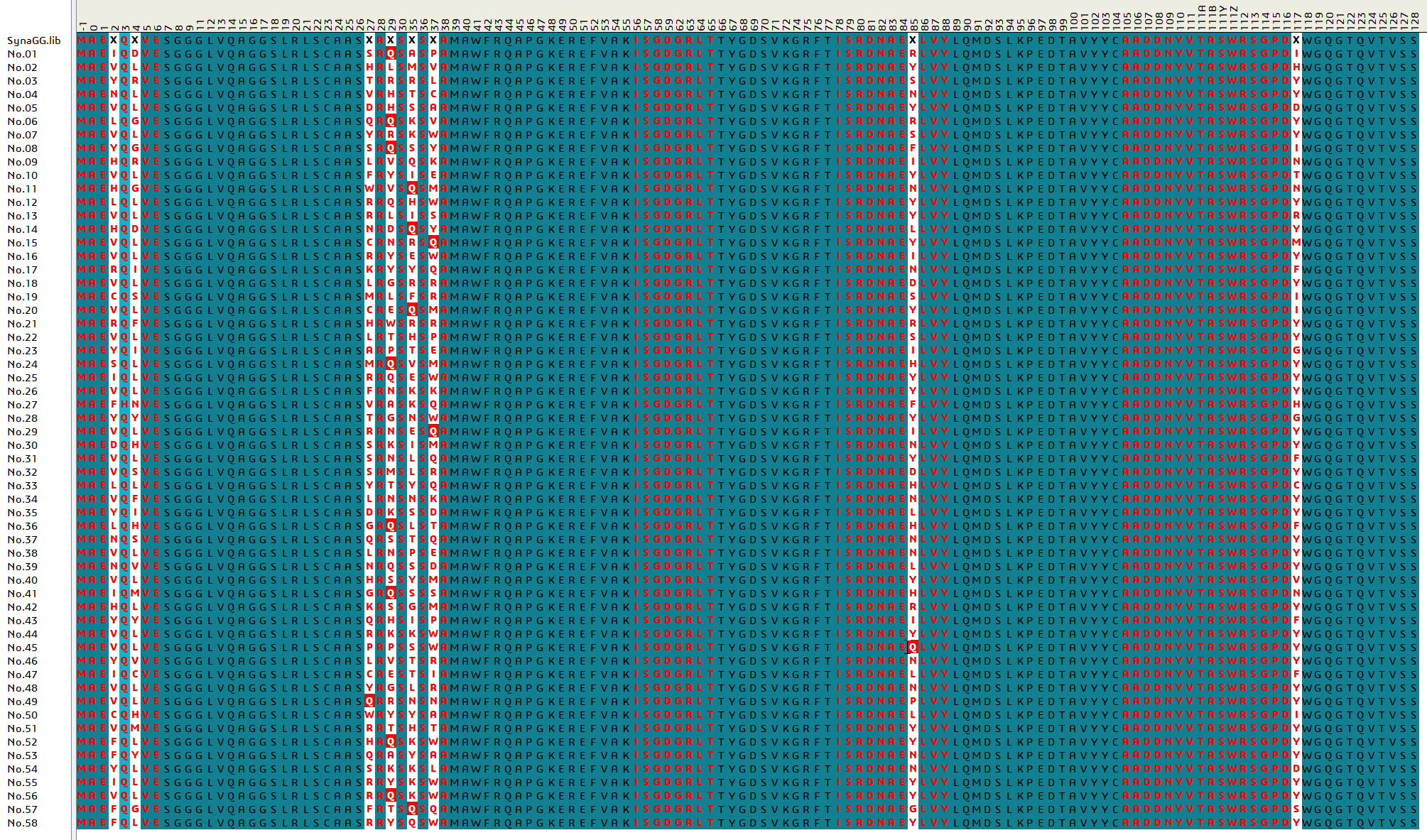


**Supplementary Figure S2. Amplified library phage ELISA and single colony analysis.** (a) The amplified phage library after each round of panning was used to confirm the binding reactivity by using phage ELISA. Ori denotes original phage library before the panning. (b) The binding activity of randomly selected sdAb clones to AFB1-BSA was examined using ELISA.


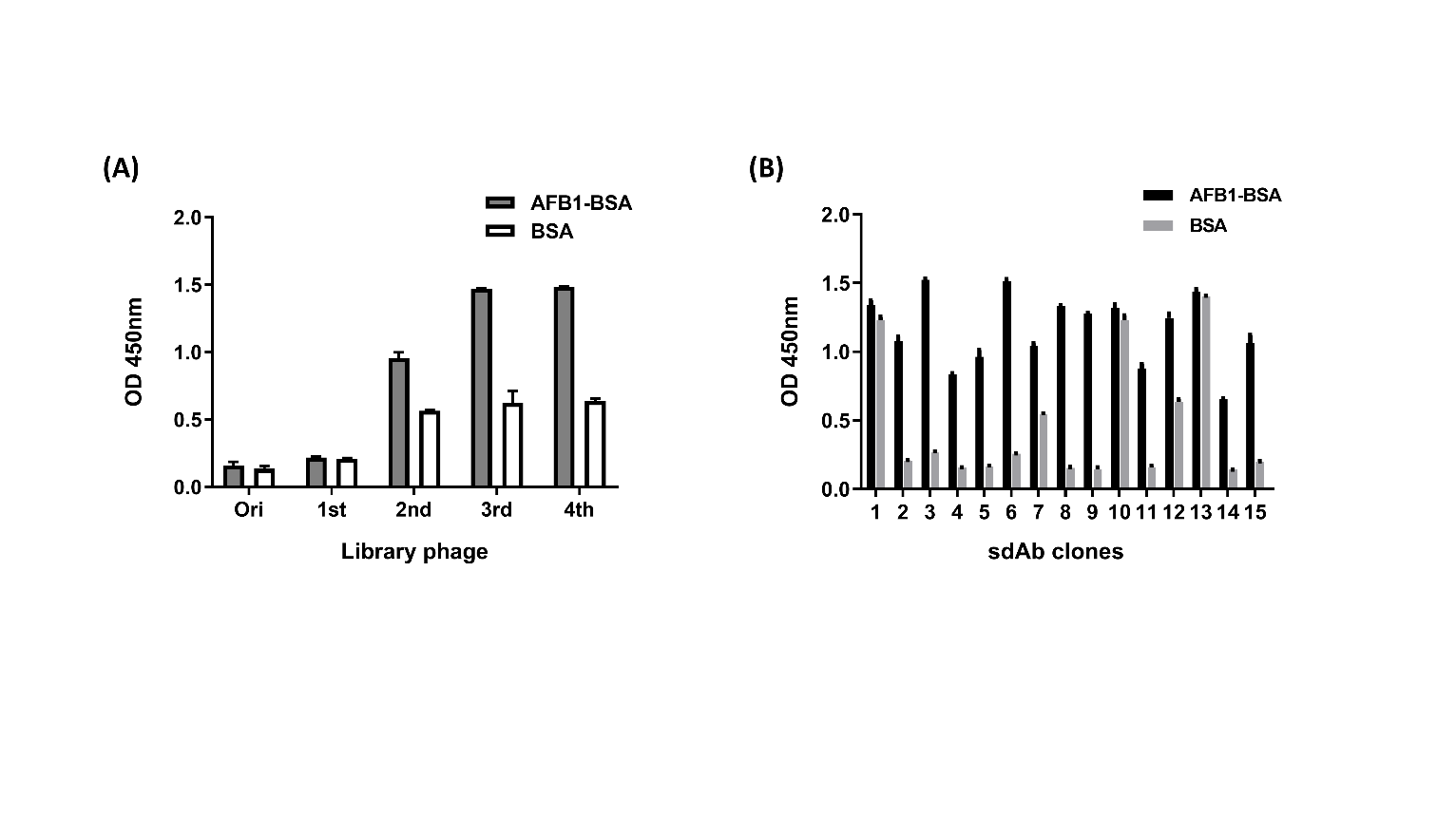


**Supplementary Figure S3. The purified nanobody proteins were analyzed by SDS-PAGE.** Four nanobody clones were induced by IPTG for protein expression and subsequently purified by Ni^2+^-charged sepharose. After analyzing by SDS-PAGE under the reducing condition, purified nanobodies were visualized through Coomassie blue staining. Red arrows indicate the molecular weight of the nanobody (approximately 15 kDa). M denotes protein ladder, S represents the cell lysate after IPTG induction, and B means the sepharose after incubation with cell lysate. In the last lane, P denotes the purified nanobody protein eluted from the sepharose.


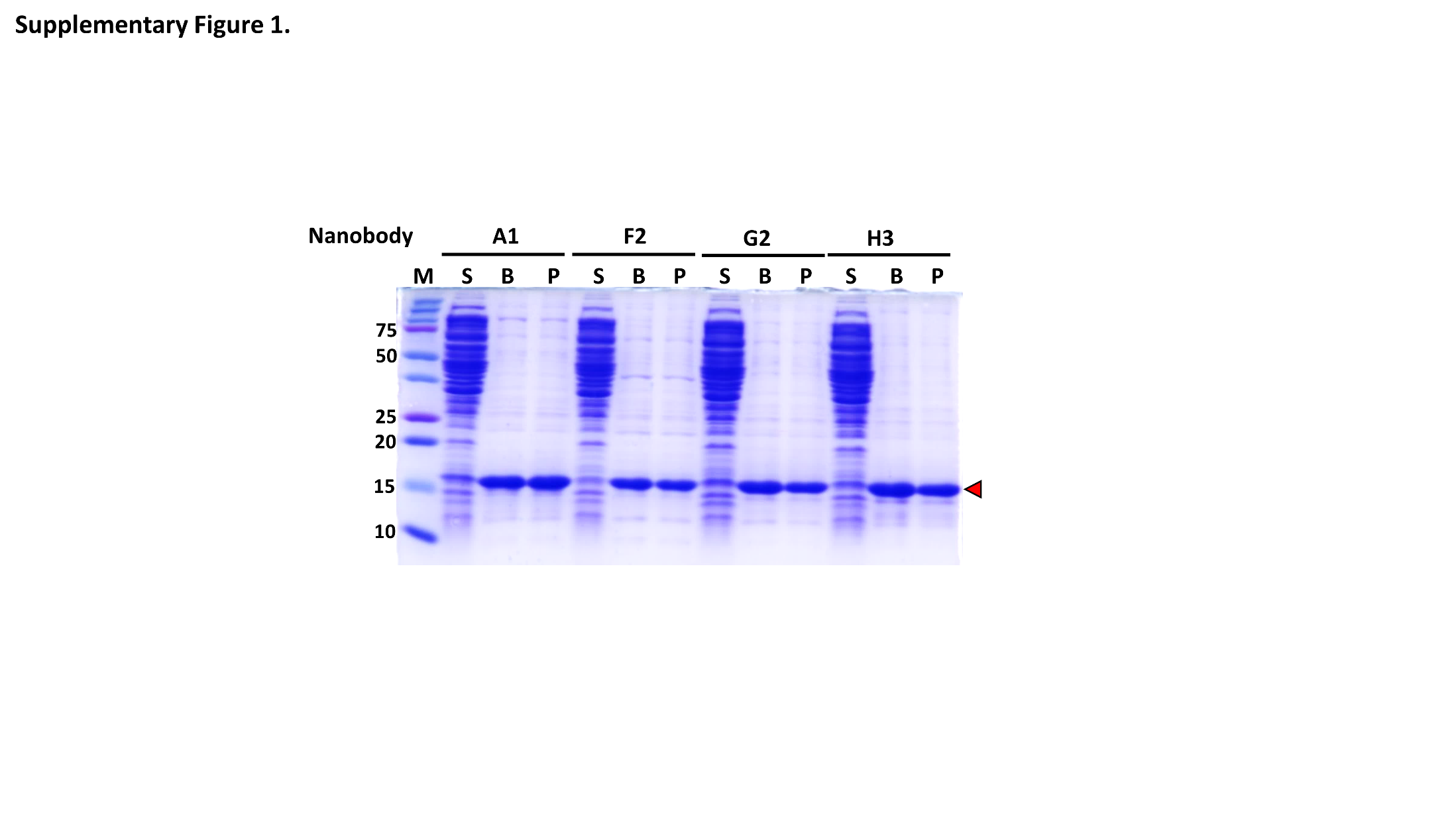

Supplement: Supplementary file 1 — Additional file 1: Supplementary Fig. S1. The 58 sequences with complete NNK substitution on 8 mutagenesis positions in the SynaGG library. Supplementary Fig. S2. Amplified library phage ELISA and single colony analysis. Supplementary Fig. S3. The purified nanobody proteins were analyzed by SDS-PAGE. Please check additional file if captured correctly. Correct, but the fourth affiliation needs to be revised to match the manuscript. Revise from: The Ph.D. Program for Medical Biotechnology, College of Medical Science and Technology To: Ph.D. Program in Medical Biotechnology, College of Medical Science and Technology. [file 13036_2023_350_MOESM1_ESM.docx]
